# *TOLLIP* and *MUC5B* Genetic Variants Influence the Circulating KL6, SPD, and HE4 Levels in Interstitial Lung Abnormalities

**DOI:** 10.3390/diagnostics16213526

**Published:** 2026-10-27

**Authors:** Leslie Chavez-Galan, Ranferi Ocaña-Guzmán, Lucero Angeles Ramón-Luing, Ingrid Fricke-Galindo, Gloria Pérez-Rubio, Ramcés Falfán-Valencia, Iliana Herrera, Ivette Buendia-Roldan

**Affiliations:** Instituto Nacional de Enfermedades Respiratorias Ismael Cosío Villegas, Tlalpan, Mexico City 14080, Mexico

**Keywords:** interstitial lung abnormalities, biomarkers, genetic variants, *MUC5B*, *TOLLIP*, progression

## Abstract

**Background:** Interstitial lung abnormalities (ILAs) are alterations of lung parenchyma found in approximately 5% of individuals older than 60 years. There is growing interest in identifying early biomarkers that may facilitate diagnosis and predict disease progression. Given ILA’s biological complexity, a multidimensional approach integrating genetic susceptibility and immune dysregulation is warranted. **Methods:** We studied 87 ILA subjects and 74 subjects without changes on high-resolution computed tomography and without respiratory symptoms. We also classified ILA subjects by progression status during 5 years of follow-up: 20 progressed, and 67 did not. This study evaluated demographic characteristics, circulating soluble proteins (KL6, SPD, and HE4), T cell polarization (Th1/Th2 balance), and selected gene polymorphisms (*TERT*, *MUC5B*, *FAM13A*, *TOLLIP*, and *DSP*). **Results:** Patients with ILA were predominantly male and showed significant abnormalities in pulmonary function tests; they had higher circulating SP-D levels (78 ± 44 versus 43 ± 31 ng/mL, *p* < 0.0001). Genetic variants significantly influence circulating levels; the median KL6 levels were higher in patients and controls carrying the *TOLLIP* rs5743890 TT genotype (0.27 ng/mL [0.01–0.71 ng/mL]) than in those with the TC genotype (0.01 ng/mL [0.00–0.19 ng/mL]). The SPD levels according to the *MUC5B* rs35705950 genotypes were lower in controls and patients carrying the common genotype GG (48.66 ng/mL [27.49–80.40 ng/mL] and 56.91 ng/mL [32.23–90.22 ng/mL]) than subjects with GT + TT genotypes (83.09 ng/mL [56.52–139.72 ng/mL], and 93.70 ng/mL [57.77–154.38 ng/mL]) and HE4 levels were different when the patients and controls carrying the *MUC5B* rs35705950 GG (2.43 pmol/mL [1.56–3.22 pmol/mL], and 2.43 pmol/mL [1.65–3.29 pmol/mL]) were compared to those with the GT genotype (1.56 pmol/mL [1.21–1.97 pmol/mL], and 1.48 pmol/mL [1.08–2.09 pmol/mL]). No difference emerged when we divided ILAs by progression. The Th1/Th2 ratio does not differ between ILAs and controls, even after polyclonal stimulation. **Conclusions:** Our findings provide relevant insight into the relationship between genetic background and circulating levels.

## 1. Introduction

Interstitial lung abnormalities (ILAs) are lung parenchymal alterations found in about 5% of individuals older than 60 years. According to the recent American Thoracic Society Clinical Statement, ILAs are defined as nondependent bilateral parenchymal abnormalities detected on CT, including ground-glass opacities, reticular abnormalities, architectural distortion, traction bronchiectasis, and/or honeycombing, that affect more than 5% of any lung zone by visual assessment. However, these individuals do not meet diagnostic criteria for interstitial lung disease (ILD) [1,2,3].

Since 2015, our research group has followed a longitudinal aging cohort, and we reported an ILA prevalence of 9%, with clinical characteristics comparable to those described in other populations [4]. Interest is growing in identifying early biomarkers that may facilitate diagnosis and, more importantly, predict disease progression. Previous studies have reported microRNA dysregulation associated with cellular senescence, a pro-inflammatory microenvironment characterized by CD4^+^ T cell hyperactivation with a Th1-predominant profile, and genetic susceptibility linked to specific HLA alleles [5,6]. Despite these advances, no validated biomarkers currently exist to identify or stratify risk in individuals with ILA.

Some longitudinal studies have shown that a subset of individuals with ILA exhibits radiologic progression over 3–5 years of follow-up, supporting the concept that ILAs may represent an early or subclinical stage within the spectrum of diffuse interstitial lung diseases (DILDs) [1]. However, the pathogenic mechanisms underlying progression remain poorly understood, and no biomarkers have been established to identify those individuals at the highest risk of progression. Although tomographic changes may signal disease evolution, ILAs are frequently detected incidentally, limiting opportunities for timely risk assessment.

In addition, genome-wide association studies (GWAS) have further supported a genetic contribution to ILA. The promoter variant rs35705950 in the Mucin 5 B (*MUC5B*) gene has been strongly associated with ILAs (*p* = 2.6 × 10^−27^) and subpleural ILAs (*p* = 1.6 × 10^−29^) [7]. Moreover, several loci previously implicated in idiopathic pulmonary fibrosis (IPF), including Dipeptidyl peptidase 9 (*DPP9*), Desmoplakin (*DSP*), Family with sequence similarity 13 member A (*FAM13A*), Isovaleryl-CoA dehydrogenase *(IVD*), and *MUC5B*, were associated with ILAs [7]. Notably, these studies did not specifically address the relationship between these genetic variants and radiologic progression, leaving an important knowledge gap.

Given ILA’s biological complexity, the present study aimed to evaluate a multidimensional approach that integrates genetic susceptibility and immune dysregulation by examining T cell polarization (Th1/Th2 balance) and selected gene polymorphisms (Telomerase reverse transcriptase [*TERT*], *MUC5B*, *FAM13A*, Toll-interacting protein [*TOLLIP*], and *DSP*). We also sought to identify biomarkers reported in interstitial lung diseases, including inflammation- or fibrosis-related circulating soluble proteins (Krebs von den Lungen-6 [KL6], surfactant protein D [SPD], and human epididymis protein 4 [HE4]) in individuals with ILA.

## 2. Materials and Methods

### 2.1. Study Population

We studied 87 subjects with a diagnosis of ILA in accordance with the latest definition of the American Thoracic Society Clinical Statement [1], and 74 subjects without changes on high-resolution computed tomography and without respiratory symptoms, who are participating in the “lung aging cohort” of the National Institute of Respiratory Diseases in Mexico City. We excluded subjects with ILA who did not have 5 years of follow-up in our cohort.

We classified ILA subjects by progression status over 5 years of follow-up. Progression is considered when the patient presented two of the following conditions: (1) decline of pulmonary function tests of either FVC > 5%, DLCO > 10%, or reduction of 50 m in the 6 min walking test, (2) initiation or worsening of respiratory symptoms, or (3) increase of >10% of lesions compared with previous computed tomography or the appearance of new lesions such as honeycombing or traction bronchiectasis. In our cohort, 20 ILAs progressed, and 67 did not.

The Scientific and Ethics Committee of the National Institute of Respiratory Diseases approved this study (C27-23) on 20 June 2023, and all subjects provided written consent.

### 2.2. Peripheral Blood Mononuclear Cells

Peripheral blood mononuclear cells (PBMCs) were isolated by standard Lymphoprep^TM^ (Accurate Chemical-Scientific, Westbury, NY, USA) density gradient centrifugation (30 min at 500× *g* and 16 °C) and cryopreserved until use. Serum samples were separated from peripheral blood by centrifugation at 4000 rpm at 4 °C for 20 min, then collected and stored at −80 °C until use.

Briefly, after centrifugation, the interface cells were collected, washed three times in phosphate buffer solution 1X, and counted in a Neubauer chamber; cellular viability was determined by a trypan blue dye exclusion method, and cells were cryopreserved in a solution of fetal bovine serum 90% (FBS, GIBCO, Grand Island, NY, USA) and 10% DMSO (Sigma-Aldrich, St. Louis, MO, USA) in liquid nitrogen storage.

We moved the cryovial from liquid nitrogen into a 37 °C water bath until it was nearly thawed (2–5 min). We transferred the thawed cells into a centrifuge tube containing RPMI-1640 culture medium (GIBCO) supplemented with 2 mM L-glutamine (GIBCO), 100 μg/mL streptomycin, 100 IU/mL penicillin, and 10% FBS (GIBCO), then centrifuged the cell suspension to obtain the cell pellet. We determined cell viability by the trypan blue exclusion assay after thawing; we required a minimum of 70% viability to proceed with cell processing.

### 2.3. PBMCs Stimulation

Firstly, 0.5 × 10^6^ of PBMCs from controls and ILA were plated in supplemented RPMI-1640 culture medium. PBMCs were stimulated with 1 μg/mL of monoclonal antibodies (mAbs), ultra-leaf-purified anti-CD3 and anti-CD28 (BioLegend, San Diego, CA, USA), or with a mixture of 1X phorbol-12-myristate-13-acetate with ionomycin (Thermo Fisher Scientific, Carlsbad, CA, USA), or with an IgG1 isotype control (I.C.). We maintained the culture for 72 h at 37 °C in a 5% CO_2_ atmosphere. Before harvest, we added monensin (0.002 mM, BioLegend) to the cell culture 2 h before harvest, then recovered the cells.

### 2.4. Flow Cytometry Analysis

We prepared PBMCs to evaluate cell-surface marker expression using mAbs against CD2, CD3, and CD4. For intracellular staining, we used an mAbs cocktail against T-bet and GATA-3. Briefly, after extracellular staining, we permeabilized cells with BD Cytofix/Cytoperm™ buffer (BD Biosciences) for 20 min at 4 °C, then incubated them with the intracellular mAbs for 30 min at 4 °C in the dark. We stained and acquired Fluorescence Minus One conditions in parallel to identify background staining; we excluded dead cells using the Zombie Red Fixable viability kit (BioLegend). We acquired the data on a FACSAria-II flow cytometer (BD Biosciences, San Jose, CA, USA) using FACSDiva 6.1.3 software. In each condition, we acquired at least 50,000 events per sample. We analyzed the flow cytometry data file using FlowJo™ (FlowJo, LLC, Ashland, OR, USA) v10.6.1 and reported the frequency profile of the CD4+ T cell subpopulation (Th1 and Th2); we also calculated the Th1/Th2 ratio.

As an analysis strategy, we first used FSC data to exclude doublets (FSC-A vs. FSC-H) and selected viable events (negative for the viability marker). We then selected CD2+CD3+CD4+ T cells to evaluate Tbet and GATA3 expression, using the FMO as a control to define each gate, and determined the frequency of CD4+ GATA+ and CD4+ Tbet+ cells.

### 2.5. Blood Sampling and DNA Isolation

We had 137 samples (69 from the ILA group and 68 from controls) available for DNA isolation and genotyping analyses. We extracted genomic DNA from blood cells using standard protocols from 10 mL of peripheral blood with the Blood DNA Preparation–Solution Kit (Jena Bioscience, Jena, Germany). The DNA was quantified using a NanoDrop 2000 spectrophotometer (Thermo Scientific, Wilmington, DE, USA) via UV/Vis spectrophotometry, using the 260/280 and 260/230 ratios to assess sample purity (acceptable ranges: 1.8–2.0 and >2, respectively). We then stored the samples at −80 °C until further use. We then adjusted each sample to 15 ng/µL for genotyping.

### 2.6. Single-Nucleotide Variants Selection

We genotyped six single-nucleotide polymorphisms (SNPs) in five genes (*MUC5B* rs35705950, *FAM13A* rs2609255, *TERT* rs2736100, *DSP* rs2076295, *TOLLIP* rs111521887, and rs5743890) using TaqMan-specific probes (Applied Biosystems, Carlsbad, CA, USA). The rs35705950 gene promoter variant in *MUC5B* and five intronic SNVs are in genes selected for their importance in pulmonary diseases and lung function. According to eQTL studies in HaploReg v4.2 [8], intronic variants affect regulatory motifs and gene expression [9].

We performed the PCR in a 25 μL reaction mixture containing 2 μL of DNA (30 ng) and 12.5 μL of 2× PCR Master Mix (Applied Biosystems, Waltham, MA, USA). The PCR conditions were 2 min at 95 °C, followed by 40 cycles of 15 s at 95 °C and 1 min at 60 °C. Allele discrimination was performed using SDS (Sequence Detection System) v. 1.4 (Applied Biosystems, San Francisco, CA, USA). This reliable, widely used method genotypes single-nucleotide variants.

### 2.7. Evaluation of Serum Biomarkers by ELISA

Serum levels of SP-D and HE-4 (R&D Systems, Minneapolis, MN, USA), and KL-6 (MyBioSource, Inc., San Diego, CA, USA) were quantified by ELISA following the manufacturer’s protocols. The optical density at 450 nm was measured using a microplate reader spectrophotometer (Imark, Bio-Rad, Hercules, CA, USA). We calculated all measurements by interpolation using the corresponding standard curve. Table 1 shows details of the antibodies and ELISA kits used in this study.

### 2.8. Statistical Analysis

We presented descriptive data as frequencies with percentages or as means and standard deviations. We performed univariate analyses of baseline variables using *t*-tests, Mann–Whitney U tests, chi-squared tests, or Fisher’s exact test, as appropriate for the data. We tested normality with the Kolmogorov–Smirnov test; we considered *p* < 0.05 significant. We performed ROC analysis for each biomarker to identify cut-points to differentiate progression. All analyses were performed using GraphPad Prism (Windows or Mac, Version 11).

We assessed genetic associations in PLINK v1.07 [10] and used logistic regression to adjust for covariates that differed significantly between comparison groups. The statistical tests were conducted in RStudio 2026.04.0+526 [11]. We set *p* < 0.05 as statistically significant in all cases.

## 3. Results

The tables below show the clinical characteristics of the different groups. Table 2 shows that, as expected, patients with ILA were predominantly male and, at the time of diagnosis, showed significant abnormalities in pulmonary function tests.

Table 3 shows the characteristics of API progressors and non-progressors separately, and the clinical data are essentially the same.

### 3.1. Serum Biomarkers

**Figure 1:**
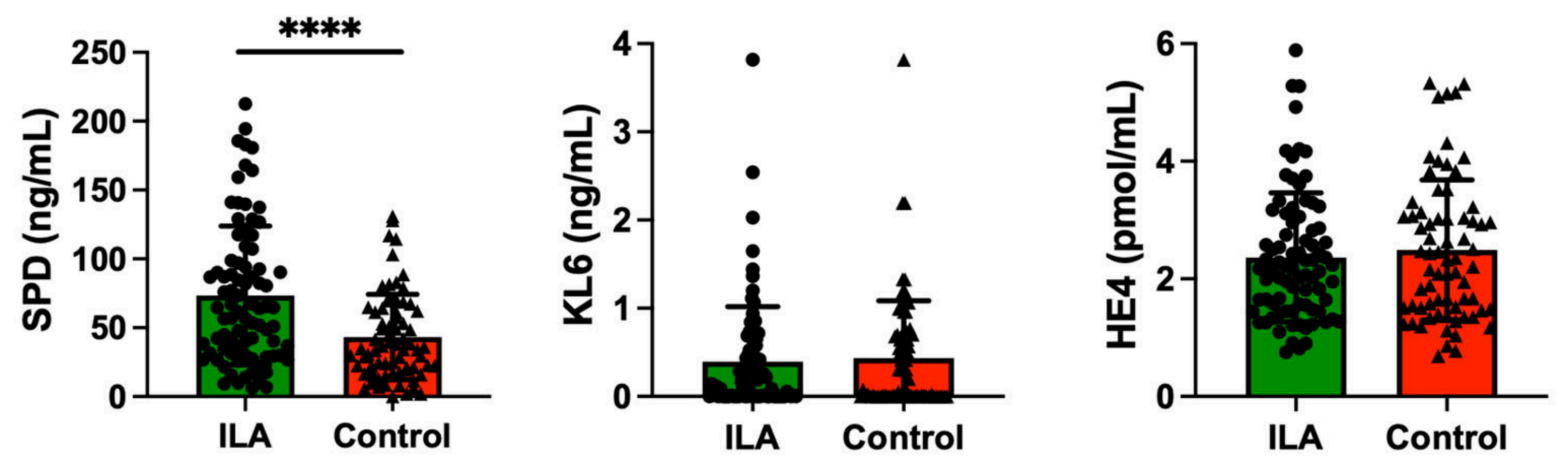
ILA patients show high SPD serum levels. We measured SPD, KL6, and HE4 by ELISA in serum from patients with interstitial lung disease (ILD) and healthy controls. Data are presented as mean ± standard deviation, and each symbol represents an individual patient; therefore, the maximum and minimum values are shown. Statistical comparisons were performed using the Mann–Whitney U test; **** *p* < 0.0001.

ILA patients had higher serum SPD levels than the control group (mean 73 ± 50 ng/mL versus 43 ± 31 ng/mL, *p* < 0.0001); however, progression status did not differ. Serum levels of KL6 and HE4 did not differ between ILA and control (Figure 1).

When we analyzed differences between ILA groups and performed ROC analysis, we did not find adequate differences or area under the curve for any biomarker to differentiate progression from non-progression. Table 3 shows mean level values. In summary, the data suggest that high SPD levels can help differentiate a healthy subject from an ILA patient, but this molecule is not involved in lung damage progression.

### 3.2. Circulating Th1/Th2 Lymphocytes

Adequate activation of pro- and anti-inflammatory CD4+ T cells is fundamental to avoiding damage induced by uncontrolled inflammation. The nuclear factors Tbet and GATA3 are activated, favoring Th1 and Th2 phenotypes, respectively. We then evaluated the Th1 and Th2 profiles of CD4+ T cells by flow cytometry.

**Figure 2:**
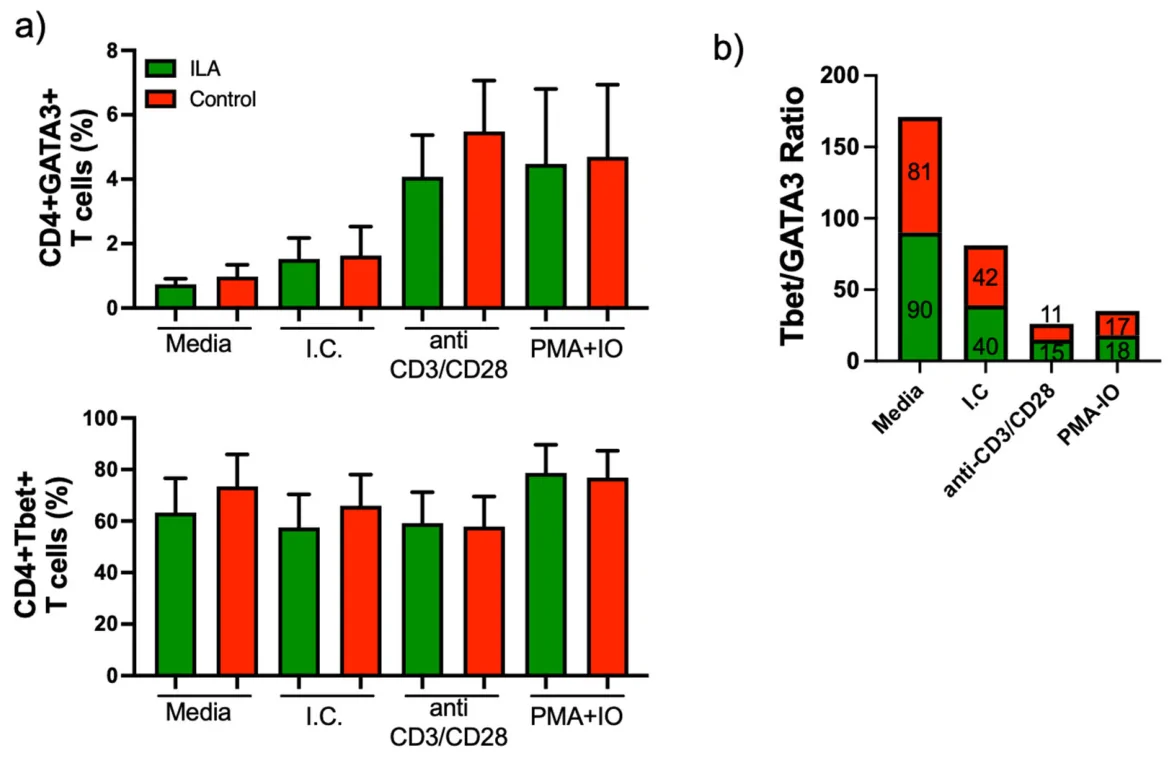
ILA patients show high SPD serum levels. We stimulated mononuclear cells from interstitial lung abnormalities (ILA) and control patients for 72 h with polyclonal stimuli using anti-CD3 and anti-CD28 monoclonal antibodies (1 µg/mL) or Phorbol Myristate Acetate and Ionomycin (PMA/IO, 1×). We used an unstimulated condition (media) and an isotype control (I.C.) as controls. (**a**) We measured the frequency of CD4+ T cells positive for GATA3 (up) or Tbet (down). (**b**) We calculated the Th1/Th2 ratio by dividing the mean frequency of Tbet by that of GATA3; we then graphed and indicated the mean ratio. Data are presented as mean ± standard deviation (n = 8 per group).

At baseline (media), both groups had a major Th1 (Tbet+) and near-null Th2 (GATA3+); however, after polyclonal stimulation, Th2 increased, and ILA and controls showed a similar pattern (Figure 2a). As expected, the Th1/Th2 ratio did not differ between groups, although it was lower under polyclonal stimulation (Figure 2b).

### 3.3. Genetic Association Analysis

We adjusted genetic association analyses for ILA risk for sex using logistic regression. The GT genotype of rs2076295 in the *DSP* gene was associated with a low risk (*p* = 0.04, OR = 0.35, 95% CI = 0.12–0.95). For the variant rs35705950 located in *MUC5B*, the GT genotype offers an increased risk for ILA (*p* = 0.03, OR = 4.64, 95%CI =1.21–22.80); this finding is confirmed in the dominant association model (GT + TT vs. GG). We found that subjects harboring one or two copies of the allele T (GT + TT) have an increased risk of ILA (*p* = 0.01, OR = 6.03, 95%CI = 1.67–28.82) when adjusting for sex compared with homozygotes for the common allele (GG). Interpret these results with caution, as the small sample size may underpower the association findings.

The logistic regression models for the remaining genetic variants were not statistically significant. In addition, no genetic variant was associated with ILA progression in the patient group.

### 3.4. Influence of Genetic Variants on the Levels of the Biomarkers

We wondered whether independent genotypes and a dominant association model for *DSP*, *FAM13A*, *MUC5B*, *TERT*, and *TOLLIP* variants influenced plasma levels of the KL6, SPD, and HE4 biomarkers.

**Figure 3:**
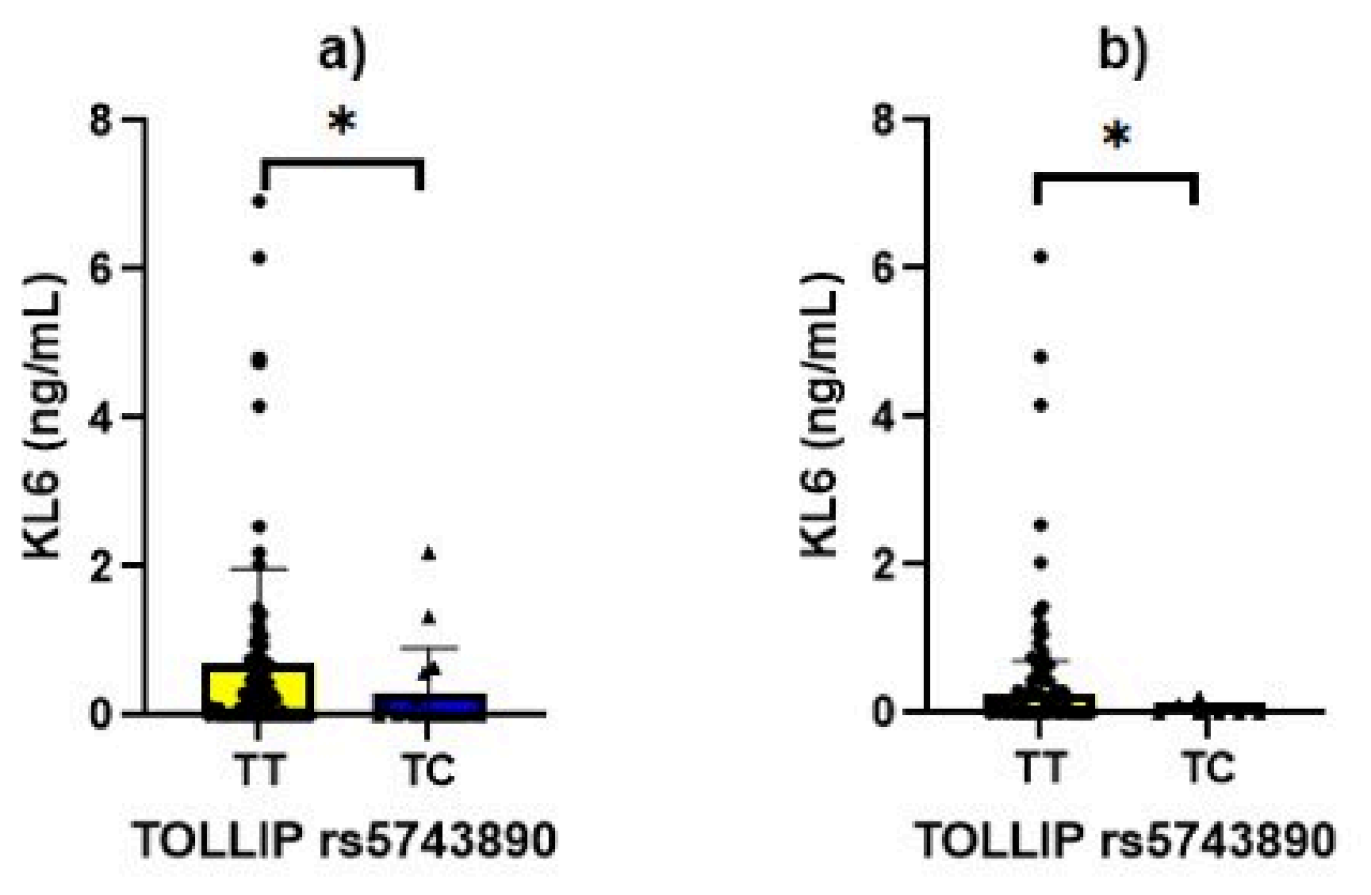
Differences in KL6 levels according to the *TOLLIP* rs5743890 genotypes when (**a**) patients and controls were included (n = 137, * *p* = 0.041) and when (**b**) only patients were analyzed (n = 69, * *p* = 0.10)—Mann–Whitney U test.

We observed that the median KL6 levels were higher in patients and controls carrying the TOLLIP rs5743890 TT genotype (0.27 ng/mL [0.01–0.71 ng/mL]) than in those with the TC genotype (0.01 ng/mL [0.00–0.19 ng/mL]; Figure 3a). The finding was also replicated when only patients were included in the comparison (TT, 0.25 ng/mL [0.01–0.69 ng/mL] vs. TC, 0.00 ng/mL [0.00–0.06 ng/mL], Figure 3b).

**Figure 4:**
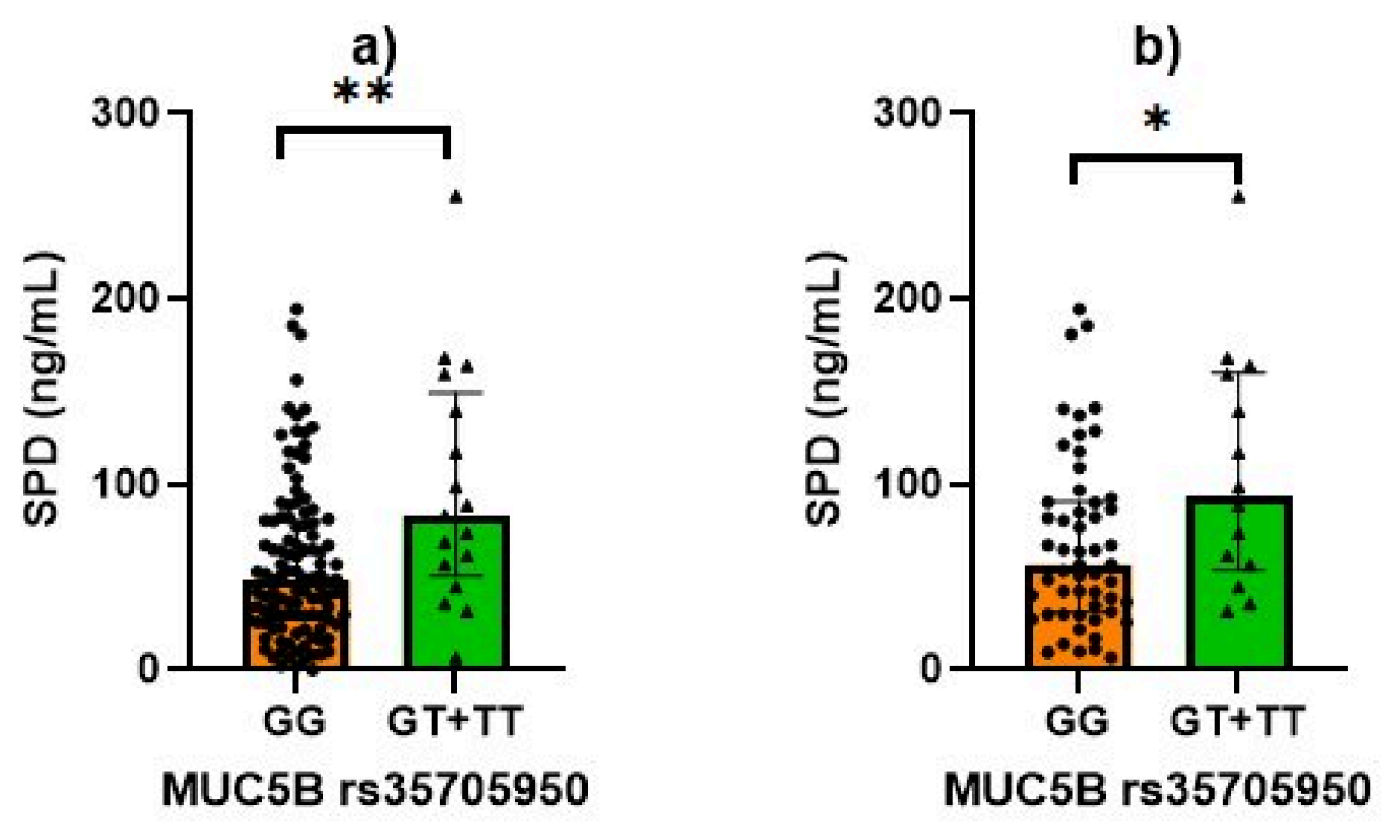
Differences in SPD levels according to the *MUC5B* rs35705950 genotypes when (**a**) patients and controls were included (n = 137, ** *p* = 0.006) and when (**b**) only patients were analyzed (n = 69, * *p* = 0.033)—Mann–Whitney U test.

Moreover, we observed differences in SPD and HE4 levels by *MUC5B*rs35705950 genotype. The SPD levels were lower in controls and patients carrying the common genotype GG (48.66 ng/mL [27.49–80.40 ng/mL] and 56.91 ng/mL [32.23–90.22 ng/mL]) than subjects with GT + TT genotypes (83.09 ng/mL [56.52–139.72 ng/mL], and 93.70 ng/mL [57.77–154.38 ng/mL]) (Figure 4a and Figure 4b, respectively).

**Figure 5:**
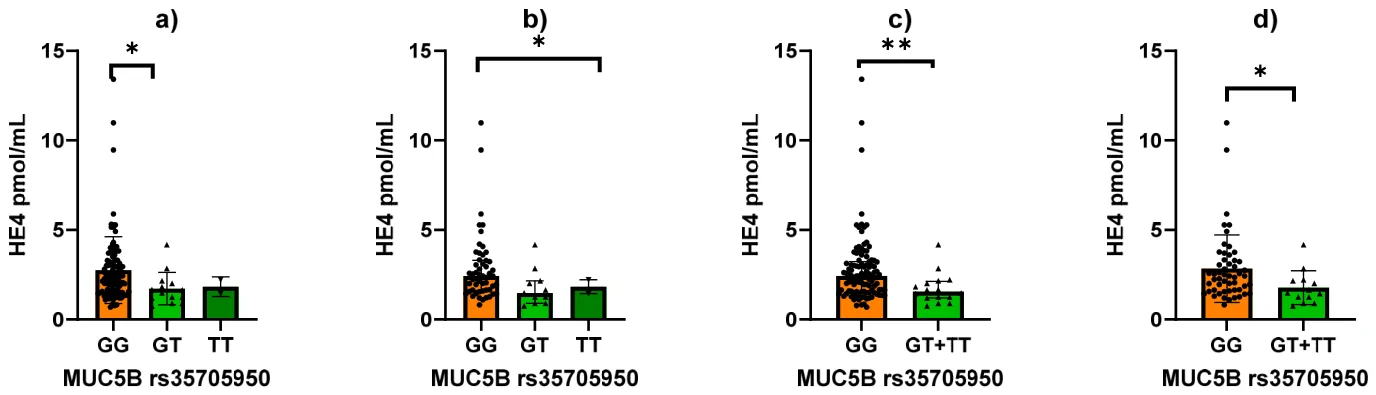
Differences in HE4 levels according to the *MUC5B* rs35705950 genotypes when (**a**,**c**) patients and controls were included (n = 137, (**a**) *p* = 0.017 [all], *p* = 0.018 [GG vs. GT], and (**c**) *p* = 0.004) and when (**b**,**d**) only patients were analyzed (n = 69, (**b**) *p* = 0.041 [all], *p* = 0.053 [GG vs. GT], and (**d**) *p* = 0.012). Only significant *p*-values (* *p* < 0.05, ** *p* > 0.004) are shown. Mann–Whitney U test (**c**,**d**) and Kruskal–Wallis tests with Benjamini–Hochberg correction (**a**,**b**).

Meanwhile, the HE4 levels were different when the patients and controls carrying the *MUC5B* rs35705950 GG (2.43 pmol/mL [1.56–3.22 pmol/mL], and 2.43 pmol/mL [1.65–3.29 pmol/mL]) were compared to those with the GT genotype (1.56 pmol/mL [1.21–1.97 pmol/mL], and 1.48 pmol/mL [1.08–2.09 pmol/mL]). As shown in Figure 5a and Figure 5b, respectively, we identified a statistical trend (*p* = 0.053), and with GT + TT genotypes (1.56 pmol/mL [1.24–2.06 pmol/mL] and 1.48 pmol/mL [1.26–2.16 pmol/mL]) (Figure 5c and Figure 5d, respectively).

The remaining comparisons between genotypes and protein levels were not statistically significant.

## 4. Discussion

In this study, our findings provide insight into the relationship between genetic background and circulating levels of KL6, SPD, and HE4, molecules previously associated with other ILDs [12,13,14].

After 5 years of follow-up, our Mexican aging cohort showed that male sex was more frequently associated with lower DLCO values, a reduced DLCO/VA index, and shorter distances on the 6 min walk test. These findings are consistent with our previous 5-year follow-up report [3]. In contrast to other ILA cohorts, in which more than half of individuals exhibited clinical worsening or decline in pulmonary function over 5 years [2,15,16,17], only 23% of our patients met the criteria for progression. This lower progression rate may reflect population-specific factors, environmental exposures, genetic background, or differences in follow-up strategies; therefore, we need to define specific criteria to classify progression in ILAs or assess whether criteria for progressive pulmonary fibrosis apply to this group.

A previous study showed that individuals with ILAs display hyperfunctional CD4^+^ T cells accompanied by loss of the inhibitory receptor KLRG1, a mechanism that may favor sustained inflammatory activation [18]. In this study, the Th1/Th2 ratio remained stable even after polyclonal stimulation. Notably, both ILA patients and controls showed increased Th2 frequency, whereas Th1 changes were modest. These observations suggest that the pro-inflammatory microenvironment described in ILAs may not be driven solely by Th1 expansion, but rather by impaired expansion of CD4+GATA3+ T cells. In this context, loss of immune regulation, rather than excessive Th1 polarization per se, may contribute to persistent low-grade inflammation. Further mechanistic studies are needed to clarify the specific role of CD4+ T cell dysfunction in ILA pathophysiology and progression, enabling a better approach to patient care.

Given that diverse pathologies have been associated with ILA [19,20], this imbalance in T cell function may extend to other systems, particularly those changes observed in aging, a major risk factor for ILA. Recently, data reported that aging modifies the Th1/Th2 profile, affecting functional recovery in aging populations [21]. Thus, inflammation is a process present in both aging and ILA; circulating levels of inflammatory mediators such as C-reactive protein, interleukin-6, or tumor necrosis factor receptors have been associated with ILA, but these mediators are non-specific to ILA; they overlap with the characteristic low-grade inflammation of aging called inflammaging, which has been associated with morbidity and mortality [22,23]. In summary, although no ILA-specific immune signature has yet been defined, the predominant inflammatory profile supports the concept that ILA arises under a context of persistent immune activation and epithelial stress.

In the present study, we focused on SPD, KL6, and HE4 because they are well associated with fibrotic ILD. Although none of these molecules differentiated progressive from stable ILA, the overall ILA group showed significantly higher serum SPD levels than controls. Elevated SP-D concentrations have been associated with ILD occurrence, progression, acute exacerbations, and mortality, particularly in idiopathic pulmonary fibrosis (IPF), where higher SP-D levels correlate with progressive disease [24,25].

Thus, although SPD did not discriminate progression status in our cohort, its elevation in ILA suggests the presence of early epithelial injury or alveolar stress within this population; probably, the absence of the association with progression could be related to the extension of abnormalities on high-resolution computed tomography, or specifically to the differences in the pathophysiological mechanisms between IPF and ILA. In this context, SPD levels cannot be considered in isolation because, as we discuss below, genetic variants should be considered alongside biomarker levels. For instance, a large multi-cohort analysis reported that polygenic risk scores derived from IPF susceptibility loci are associated with both the presence and progression of ILA, but the authors indicated that multiple loci contribute to ILA susceptibility and progression [26]. As discussed recently, ILA is clinically significant, and its progression is associated with an elevated risk of developing fibrotic lung disease; therefore, progression should be viewed in a global context that includes aging and cellular senescence, genetic susceptibility, epithelial injury and dysregulated repair, and immune activation [27].

Because circulating protein levels may be influenced not only by disease activity but also by host genetic variability, we next explored whether these factors could modulate candidate biomarker levels.

We found that genetic variants in the *TOLLIP* and *MUC5B* genes significantly influence circulating KL6, SPD, and HE4 levels in patients with ILA. Both genes are located on chromosome 11 and have been previously implicated in fibrotic interstitial lung diseases. The minor allele (T) of *MUC5B* rs35705950 is strongly associated with IPF, while the minor allele (C) of *TOLLIP* rs5743890 has been linked to increased mortality and disease progression in IPF [28].

*TOLLIP* encodes an adaptor protein involved in Toll-like receptor (TLR) signaling and innate immune regulation, modulating pro-inflammatory cytokine production [29]. In our cohort, individuals with the TT genotype at rs5743890 had significantly higher circulating KL-6 levels. Together, these findings suggest that in ILA, host genetic background may influence cellular stress responses and modulate inflammation, independently of overt radiologic progression.

Similarly, the variant *MUC5B* rs35705950 influenced soluble molecule levels. Although we previously demonstrated that this polymorphism is associated with increased susceptibility to ILA in our cohort [30], we had not explored its relationship with circulating proteins. In the present study, GT + TT carriers had higher SPD levels, whereas GG carriers had higher HE4 concentrations than GT or GT + TT individuals. These findings indicate that the *MUC5B* genotype may differentially regulate epithelial-derived proteins associated with alveolar injury and fibrotic remodeling.

The biological implications of these genotype-dependent differences remain to be fully elucidated. MUC5B overexpression has been shown to enhance chemokine production, promoting inflammatory cell recruitment and mucus hypersecretion [31]. Genotype-driven alterations in mucin expression could indirectly modulate epithelial integrity, immune activation, or extracellular matrix remodeling, thereby influencing circulating biomarker levels. Molecular changes may precede measurable functional decline.

The gain-of-function *MUC5B* promoter variant (rs35705950) establishes a profoundly altered, vulnerable pulmonary epithelium long before the visual onset of IPF, reshaping the cellular proteome and destabilizing cell junctions to facilitate the systemic leakage of lung-restricted epithelial proteins into the circulation. Recent spatial proteomics and multi-omic tissue characterizations provide a deeper, four-part mechanistic framework for how this promoter variant drives subclinical epithelial stress and alters circulating biomarkers [32].

Moreover, this study has limitations. The study was conducted at a single center. Although the prevalence of ILA in our aging cohort is comparable to that reported in other populations, external validation in multicenter, ethnically diverse cohorts is necessary. The small sample size may underpower the genetic association results, and this must be considered when interpreting the results.

## 5. Conclusions

Our findings support the concept that circulating biomarkers in ILA are not solely indicators of disease activity, but may also reflect underlying genetic susceptibility. This genetic modulation could be related to single biomarkers failing to consistently discriminate progressive from stable disease across cohorts. Integrating genetic background with circulating protein profiling may therefore enhance the precision of biomarker research for risk stratification.

## Figures and Tables

**Table 1:** Details of antibodies and ELISA kits.

Antibody/Kit	Conjugate to	Catalog/Clone	Company
CD3	PE	98104/SK7	Biolegend
CD4	BV510	317443/OKT4	Biolegend
CD8	FITC	344704/SK1	Biolegend
CD2	APC Cy7	309238/TS1/8	Biolegend
GATA3	BV421	653814/16E10A23	Biolegend
Tbet	PE-Cy7	644824/4B10	Biolegend
Zombie Red™ Fixable Viability	PE-TexRed	423110	Biolegend
Ultra-LEAF™ Purified anti-human CD3 Antibody	NA	300331/HIT3a	Biolegend
Ultra-LEAF™ Purified anti-human CD28 Antibody	NA	302933/CD28.2	Biolegend
Ultra-LEAF™ Purified Mouse IgG1, κ Isotype Ctrl Antibody	NA	400165/MOPC-21	Biolegend
Ultra-LEAF™ Purified Mouse IgG2a, κ Isotype Ctrl Antibody	NA	400263/MOPC-173	Biolegend
Human SP-D Quantikine ELISA Kit	NA	DSFPD0	R&D systems
Human KL-6 ELISA Kit	NA	MBS026021	MyBioSource
Human HE4/WFDC2 DuoSet ELISA	NA	DY6274-05	R&D systems

**Table 2:** Clinical characteristics of the ILA group and controls.

Variable	ILA n = 87	Control n = 74	*p*-Value
Age, yr (±SD)	69 ± 7	68 ± 6	0.3
Male, n (%)	64 (74)	38 (51)	0.005 *
Tobacco smoking, n (%)	53 (61)	44 (59)	0.8
Body mass index (mean ± SD)	27 ± 4	28 ± 4	0.05 *
Hypertension, n (%)	27 (31)	34 (46)	0.07
Type 2 Diabetes Mellitus, n (%)	16 (18)	18 (24)	0.4
Gastroesophageal reflux, n (%)	33 (38)	26 (35)	0.7
FVC, % predicted (mean ± SD)	97 ± 17	95 ± 17	0.5
DLCO, % predicted (mean ± SD)	93 ± 19	107 ± 19	<0.0001 **
DLCO/VA index (mean ± SD)	100 ± 23	110 ± 20	0.0003 *
Meters (6MWT) (mean ± SD)	444 ± 139	500 ± 77	0.002 *
SP-D ng/mL (mean ± SD)	73 ± 50	43 ± 31	<0.0001 **
KL-6 ng/mL (mean ± SD)	0.39 ± 0.6	0.43 ± 0.6	0.6
HE4 pmol/mL (mean ± SD)	2.3 ± 1	2.5 ± 1.1	0.4

Yr: years; SD: standard deviation; FVC: forced vital capacity; DLCO: diffusing capacity of the lungs for carbon monoxide; VA: alveolar ventilation rate; MWT: minute walking test; SP-D: surfactant protein D; KL-6: Krebs von den Lungen-6; HE-4: Human epididymis protein 4. * *p* < 0.05, ** *p* < 0.0001.

**Table 3:** Clinical characteristics of patients with ILA separated as progressors and non-progressors.

Variable	ILA Progression n = 20	ILA Non-Progression n = 67	*p*-Value
Age, yr (mean ± SD)	71 ± 8	69 ± 6	0.1
Male, n (%)	10 (50)	28 (42)	0.6
Tobacco smoking, n (%)	12 (60)	41 (61)	0.9
Hypertension, n (%)	7 (35)	20 (29)	0.7
Type 2 Diabetes Mellitus, n (%)	4 (20)	12 (18)	0.9
Gastroesophageal reflux, n (%)	11 (55)	22 (33)	0.1
FVC, % predicted (mean ± SD)	95 ± 13	98 ± 19	0.5
DLCO, % predicted (mean ± SD)	86 ± 17	95 ± 20	0.1
DLCO/VA index (mean ± SD)	96 ± 33	101 ± 20	0.3
SP-D ng/mL (mean ± SD)	78 ± 44	72 ± 52	0.6
KL-6 ng/mL (mean ± SD)	0.38 ± 0.51	0.4 ± 0.6	0.9
HE4 pmol/mL (mean ± SD)	2.3 ± 1	2.3 ± 1.1	0.9

Yr: years; SD: standard deviation; FVC: forced vital capacity; DLCO: diffusing capacity of the lungs for carbon monoxide; VA: alveolar ventilation rate; SP-D: surfactant protein D; KL-6: Krebs von den Lungen-6; HE-4: Human epididymis protein 4.

## Data Availability

ClinVar Submission SCV007655913-SCV007655918.

## Abbreviations

The following abbreviations are used in this manuscript:

ILA	Interstitial lung abnormality
CT	Computed tomography
ILD	Interstitial lung disease
DILDs	Diffuse interstitial lung diseases
GWAS	Genome-wide association studies
IPF	Idiopathic pulmonary fibrosis
TERT	Telomerase reverse transcriptase
MUC5B	Mucin 5 b
DPP9	Dipeptidyl peptidase 9
FAM13A	Family with sequence similarity 13 member A
IVD	Isovaleryl-CoA dehydrogenase
TOLLIP	Toll-interacting protein
DSP	Desmoplakin
KL6	Krebs von den Lungen-6
SPD	Surfactant protein D
HE4	Human epididymis protein 4
FVC	Forced vital capacity
DLCO	Diffusing capacity of the lungs for carbon monoxide
PBMCs	Peripheral blood mononuclear cells
DNA	Deoxyribonucleic acid
SNPs	Single-nucleotide polymorphisms
VA	Volume alveolar
TLR	Toll-like receptor
